# Evolution of Tumor Metabolism might Reflect Carcinogenesis as a Reverse Evolution process (Dismantling of Multicellularity)

**DOI:** 10.3390/cancers3033002

**Published:** 2011-07-22

**Authors:** Khalid O. Alfarouk, Mohammed E.A. Shayoub, Abdel Khalig Muddathir, Gamal O. Elhassan, Adil H.H. Bashir

**Affiliations:** 1 Department of Evolution of Tumor Metabolism and Pharmacology, Hala Alfarouk Cancer Center, Khartoum 11123, Sudan; E-Mail: Adil.Bashir@Hala-alfarouk.org; 2 Department of Pharmaceutics, Faculty of Pharmacy, University of Khartoum, Khartoum 11111, Sudan; E-Mail: Shayoub2004@hotmail.com; 3 Department of Pharmacognosy, Faculty of Pharmacy, University of Khartoum, Khartoum 11111, Sudan; E-Mail: muak46@yahoo.com; 4 General Directorate of Pharmacy, Federal Ministry of Health, Khartoum 11111, Sudan; E-Mail: Gamaosma@yahoo.com; 5 Al Jawda Medical Hospital, Khartoum 11111, Sudan

**Keywords:** Warburg-effect, Crabtree-effect, Pasteur-effect, lactate symbiosis, cannibalism, reverse evolution, convergent evolution

## Abstract

Carcinogenesis occurs through a series of steps from normal into benign and finally malignant phenotype. This cancer evolutionary trajectory has been accompanied by similar metabolic transformation from normal metabolism into Pasteur and/or Crabtree-Effects into Warburg-Effect and finally Cannibalism and/or Lactate-Symbiosis. Due to lactate production as an end-product of glycolysis, tumor colonies acquire new phenotypes that rely on lactate as energetic fuel. Presence of Warburg-Effect indicates that some tumor cells undergo partial (if not complete) de-endosymbiosis and so cancer cells have been become unicellular microorganism (anti-Dollo's Law) specially when they evolve to develop cannibalism as way of metabolism while oxidative types of cells that rely on lactate, as their energetic fuel, might represent extra-endosymbiosis. Thus, at the end, the cancer colony could be considered as integrated metabolic ecosystem. Proper understanding of tumor metabolism will contribute to discover potential anticancer agents besides conventional chemotherapy.

## 1. Introduction

While yeast secrete ethanol as way of competition in the so called “Crabtree effect” [1], cancer cells produce lactate via the “Warburg-effect” to increase their fitness [2]. This raises the question: do the Warburg-effect and Crabtree-effect reflect ancestral relationships (*i.e.*, evolution of behavior)? If so, how do mammalian cells produce lactic acid during *anaerobiosis* while yeast produces ethanol? It would be great of interest to study the evolutionary trajectory of anaerobic metabolism from ethanol to lactic acid production. This is mainly because mitochondrion has been considered to be a bacterium [3], so production of ethanol will not only harm mitochondrion (antiseptic agent) but also inhibit cellular energy metabolism via inhibition of AMPK [4]. Moreover, extracellular extrusion of lactate creates slightly alkaline intracellular (pH*i*), which most probably atrophies mitochondrion as an energy producing organelle [5] but also produces a benefit because mitochondrial atrophy results in a shut-down of apoptosis [6]. Sequestration of the apoptosis pathway is a key event to develop genomic instability [7]. Thus, carcinogenesis probably represents a delicate balance between the two processes.

## 2. Carcinogenesis Key Events

**Figure 1.**
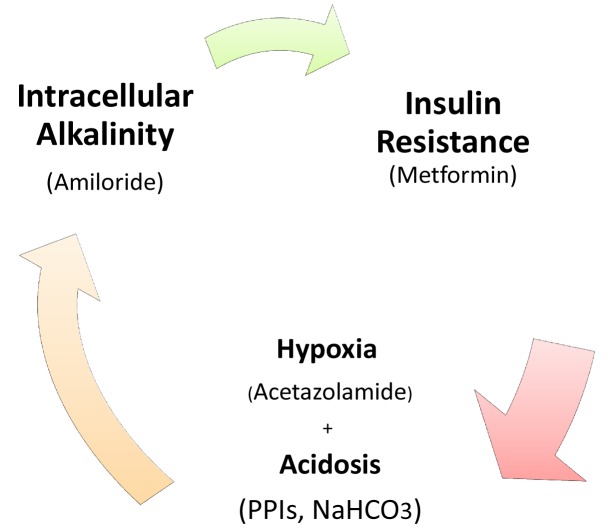
Describes the interaction between different key events of carcinogenesis, this figure represents the interaction of the possible three key events of carcinogenesis with potential antagonizing agents.

**Figure 2.**
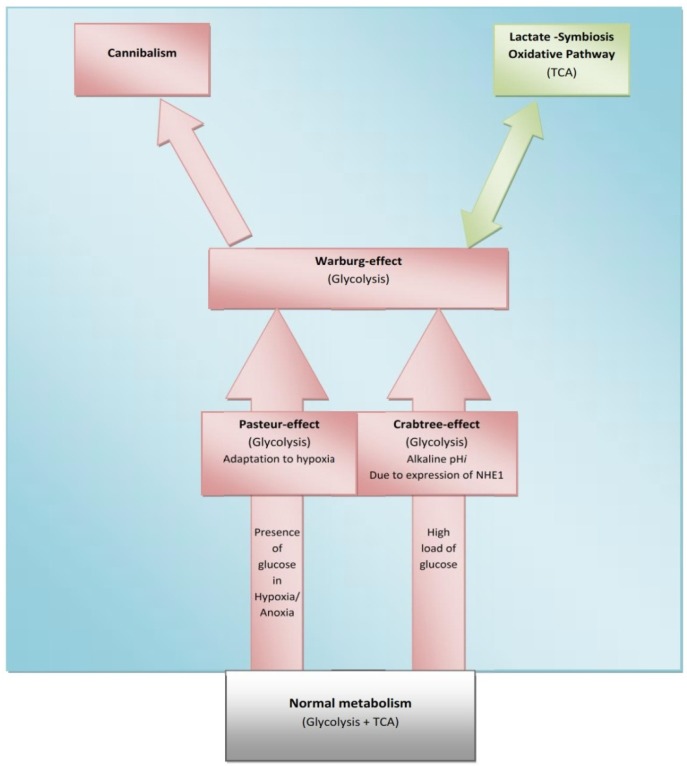
Cancer as integrated metabolic ecosystem, This model describes the possible pathways of carcinogenesis associated with metabolic phases of transformation. The green background reflects the tumor microenvironment which represents the medium on which carcinogenesis steps have occurred. Normal metabolism occurs does not create the tumor microenvironment which is why it is presented outside of the green background.

The Crabtree-effect is not confined only to yeast; it was discovered in tumors upon lactic acid production [8]. So, what are main differences between Warburg-effect and Crabtree-effect? One of the main differences that distinguish these two effects is that the Crabtree-effect occurs as a short-term adaptive mechanism after glucose-induced repression while the Warburg-effect is a long-term adaptive mechanism [9,10]. The following question would be how a high load of glucose induces the Crabtree Effect (up-regulation of glycolysis)? One of the possible answers is that a high glucose load induces expression of NHE1 [11] and so it induces (intracellular alkalinity) alkaline pH*i* and so it could be so compatible with that of the Reshkin pathway of carcinogenesis upon his novel discovery that HPV induces alkaline pH*i* as first event of phenotype transformation [12,13]. Alternatively, there is the Gatenby pathway of carcinogenesis which is based on the Warburg-Effect occurs as an adaptive strategy to face intermittent hypoxia [14,15] (Table 1). Another key event is that of the Warburg effect being a compensation to enhance and/or facilitate glucose up-take upon developing insulin resistance [16] and most probably it might explain why tumor cells respond to Metformin (Insulin sensitizing agent). Although the third key event has been recently accepted, it doesn't yet explain how the Warburg-Effect occurs as a compensation of developing tumors in tissues which have a glucose uptake independent of insulin. Interestingly, when any one of these three key events occurs, the other two then occur spontaneously (Figures 1 and 2). The conclusion that would be drawn from such key events is that a normal cell exposed to this disastrous situation would die while cancer cells switch their life-style atavistically to survive at low minimum condition and so can appear immortal. This leads to the next questions: Does cancer aim at immortality? Or immortality becomes a consequence of atavistic life-style? That is, does carcinogenesis follow Dollo's Law or not?

Targeting of these key events could be reached as follow: activity of several antitumor agents termed as Anti-Microenvironment Acidity Induced-Cancer Spite “Anti-MAICS” [2]: Where hypoxia is stimulated through the Carbonic Anhydrase inhibitor, Acetazolamide [17-19], and extracellular acidosis is targeted by (i) Proton Pump Inhibitors (PPIs) and (ii) systemic NaHCO_3_ treatment [20-23] while Amiloride [24] and PPIs again function against alkaline intracellular pH [20].

Although the Warburg-effect and its role in the creation of the tumor microenvironment is widely accepted and assumed to be a hallmark of cancer [21,25], there remains a conundrum: is it a cause of malignant transformation [26] or just an effect (sign and symptoms of cancer) [5,27]?

## 3. Evolutionary Trajectory of Cancer Metabolism

Some normal tissues under hypoxic conditions undergo anaerobic glycolysis, maximum cytoplasmic utilization of glucose, (Pasteur-effect), e.g., skeletal muscle during severe exercise [28]. Many factors can induce the Crabtree-effect as a short-term adaptive strategy e.g., high load of glucose, fructose and the changing of pH [10,29]. Interestingly, the Crabtree-effect might be adjusted to occur at an early stage of carcinogenesis [9]. Then Warburg-effect occurs, so Warburg-effect becomes as consequences and not causation, and finally cannibalism occurs during the final stages because cannibalism is a feature of secondary tumors [30]. Cannibal cells are cells types which are vulnerable to metastasis. Thus the question arises: is cannibalism a hallmark of metastasis?

**Figure 3.**
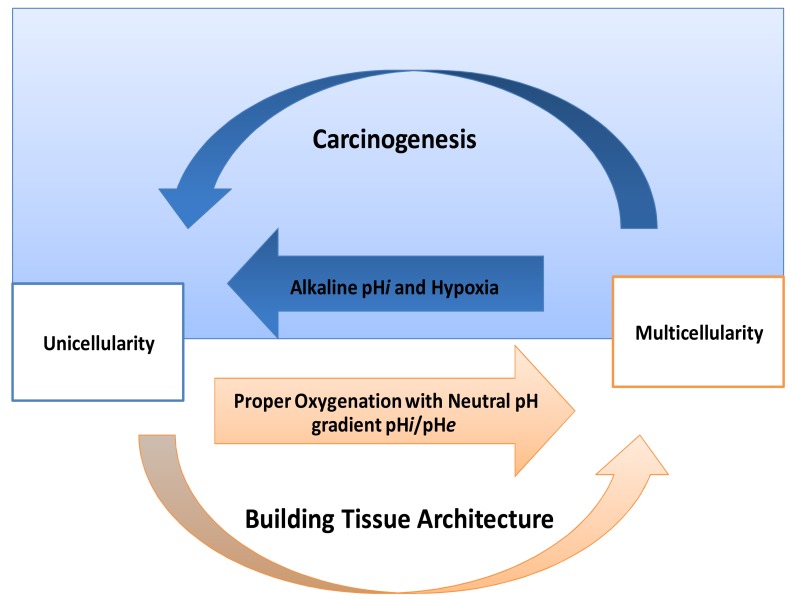
Carcinogenesis as a dismantling of multi-cellularity. Hypothetical model describes the evolutionary homeostasis between prokaryogenesis (carcinogenesis) and eukaryogenesis. The blue background reflects the tumor microenvironment which is the medium where this reverse/convergent evolution occurs.

One of the striking things in cancer is its expression of HIF-proteins [31-33]. Thus, how do normal mammalian cells survive in persistent hypoxic and/or anoxic environment? Recently, carcinogenesis as devolution process (reverse evolution) might solve this enigma *i.e.*, cancer cell becomes unicellular microorganism [34] and this mainly because carcinogenesis is a process of dismantling multicellularity [35,36]. In this regard, presence of tumor microenvironment is an important dimension to carry out such reversion (Figure 3). Does carcinogenesis represent reverse evolution (atavism) or convergent evolution? Parallel to the context, recent evidence pointed that carcinogenesis convergent evolution and losing complex features is an advantageous rather than degeneracy [37].

**Figure 4.**
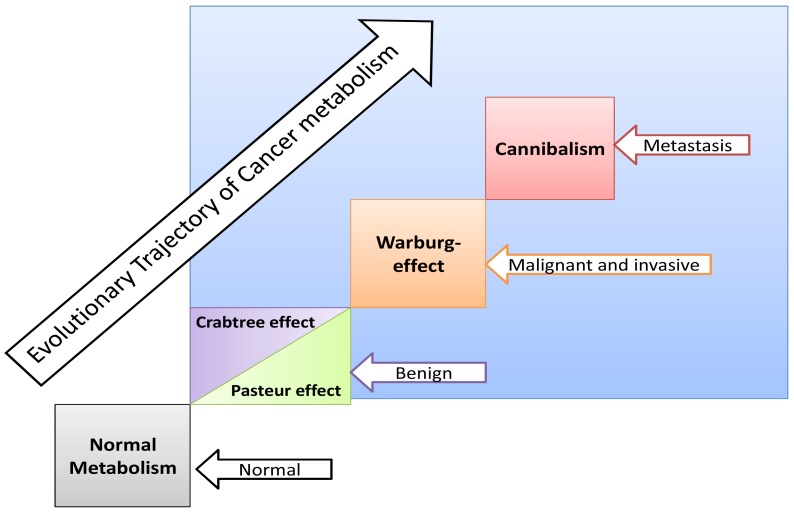
Hypothetical model describing carcinogenesis as a metabolic ladder (cascade). Bluish background represents tumor microenvironment which is the medium necessary for driving carcinogenesis.

If carcinogenesis occurs through evolutionary metabolic model, does Darwinian selection or Lamarckian inheritance follow? Based on information theory, carcinogenesis as a metabolic cascade (ladder) suggests that tumor evolution has its own evolutionary trajectory to maintain its cellular order at an energy level different from that of normal eukaryotic mammalian cells (Figure 3) [38-40]. Moreover, to maintain such order tumor cells should maintain and/or re-organize their genomic content to be in harmony with such model. Thus, genomic instability which is a feature of malignancy [25] might represent highly dynamized-ordered genomes. Does this maintenance occur Darwinian selection [7,41,42], or Lamarckian inheritance [43]? From Lamarckian point of view, this might explain how a tumor transforms from a benign into malignant phenotype upon over-expression of *hiwi* in the tumor [44], *i.e.*, the shift of the Pasteur/Crabtree-Effects into the Warburg-effect. The main remaining question is how certainly does Pasteur-effect can transform into the Crabtree-effect and to Warburg-effect? OR both Crabtree and Pasteur Effects are overlapping (Figure 4)?

## 4. Is the Warburg-Effect Glycolysis or Glutaminolysis

In tumors, if the Warburg-effect is considered to be a synonymous of an alternative strategy to generate energy in place of the oxidative phosphorylation system, it will not be of significant value because aerobic glycolysis contributes only about 10% of the total energy produced. Alternatively, if the Warburg-effect means glutaminolysis, it yields only 14%. Moreover, glycolysis together with glutaminolysis contributes only about 40%, still less than half the total energy produced [45]. Therefore, what are the advantages of the Warburg-Effect as glutaminolysis?

**Figure 5.**
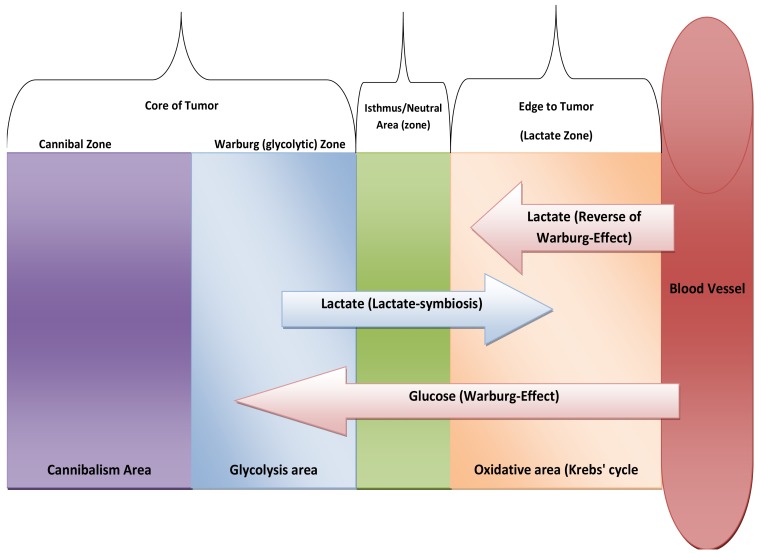
Tumor as integrated metabolic ecosystem, this model represents cancer colony as multi-layers including isthmus and/or neutral area that prevent transgress between Glycolytic phenotypic of cells and metabolic types of cells. This model is so compatible with a recent model that supported experimentally in brain tumor [54]. Because tumor cell at edge rely on lactate as energetic fuel, we suggest the term it as o “Lactate zone”. We suggest to term “*multi-layers*” and/or “*multi-zones*” to describe that tumor colony consists of several zones; each zone has distinguished energetic fuel differs from others but at the end most of these zones are in symbiotic (mutual) relationship [Cannibal zone, Warburg (glycolytic)zone, isthmus zone and Lactate zone].

It can be argued that glutaminolysis has been up-regulated in tumors mainly to serve as a pool of lactate [46] and not only for producing energy [47]. In this way, glutaminolysis represents the heart of the lactate paracrine-effect termed “lactate-symbiosis”. One of the most exciting things about lactate in cancer biology is how and why a tumor colony organizes itself as a way to divide its labor, underlined by recent evidence that some tumor cells produce energy to provide others as fuel [48]. Interestingly, it is not clear yet if this lactate is useful mainly for fuel or as a pseudo-hormone and regulatory molecule [49,50]. In addition, glutaminolysis is recruited as a precursor of nucleic acid, serine, fatty acids and cholesterol synthesis [51]. Moreover, glutaminolysis provides the cell one additional ATP molecule at the substrate level of phosphorylation. At the end, while glycolysis occurs firstly to atrophy the citric acid cycle, glutaminolysis occurs consequently to provide more lactate and meets intracellular requirements too. In other words, both glycolysis and glutaminolysis drive carcinogenesis through: (i) glycolysis occurs firstly for diminishing free radical formation through inhibition of ATP production via coupling reactions thus blocking apoptosis (atrophy of mitochondrion). After that, glutaminolysis produces lactate which is essential for extracellular acidification and provides other cells an edge through lactate as a fuel [48] (see discussion below). Thus, glutaminolysis completes the citric acid cycle mainly to provide lactate with a concomitant paralysis of coupling reaction at complexes. In conclusion, glycolysis gives the same cell: (1) energy, (2) provides lactate, (3) reduces mitochondrial atrophy (e.g., apoptosis) while glutaminolysis provides: (1) energy, (2) additional lactate, (3) stimulates fatty acid synthesis, nucleotide, *etc.* So, cancer cells recruit glycolysis and glutaminolysis for both itself and for the neighboring tumor cells and it could very well be a way of cooperation (mutualism) among tumor colony individuals or for harnessing other neighboring cells to met their requirement in a process recent discovered termed as “Reverse Warburg-Effect” [52] (Figure 5).

The tumor colony might be thermodynamically stable. Although some core cells undergo convergent and/or reverse evolution, yet surface cells might consider next evolutionary step of carcinogenesis that seen as extra-symbiosis(advanced step of eukaryogenesis) which represents losing of primitive feature as advantageous process under environmental constrain, not degeneracy, of ancestral pathway of glycolysis (microenvironment shapes tumor colony). In other words, we suggest that tumor colony is thermodynamically stable system due to evolutionary compensation. Moreover, it seems to be metastasis is an inefficient process [53] because it reflects this system to maintain its stability. Next question, does immune system might be developed to maintain thermodynamic of whole body? Parallel to the context, tumor colony represents a successful parasite originate from our bodies and so one of the possible promising strategy is negotiate with it rather than eradicate it? At the end it could be a kind of co-existence inside our body. So, it would great of interest to establish cancer treatment strategy based on negotiation rather than eradication (negotiation strategy)

## 5. Carcinogenesis as De-Endosymbiosis

The Warburg-effect means a shut-down of the Krebs cycle, which is one of the important functions of mitochondrion. The presence of this energy producing organelle is an important feature that distinguishes eukaryotes from prokaryotes [55]. So, the presence of an only partially functioning energy producing organelles might be an indicator of if some cancer cells undergo de-endosymbiosis or not. Another way to state this is that carcinogenesis is a process of towards prokaryogenesis that liberates the endosymbiotic relationship between mitochondrion and nucleus and, in this way; cancer cells actually lose endosymbiosis. The role of oxygen as a detoxifying agent by trapping H^+^ during Oxidative Phosphorylation is well known. Some anaerobic microorganisms have acquired other strategies to surmount oxygen deficiency by acquiring unique enzyme termed as Pyruvate: Ferridoxin-oxidoreductases (PFO); which trap two molecular hydrogen to produce hydrogen gas. Therefore, these microorganisms do not possess ‘mitochondrion’ but, rather, a hydrogen producing— organelle, known as a “hydrogenosome” [56]. Interestingly, the hydrogenosome and mitochondria have an ancestral relationship during their evolutionary trajectory [57-61] raising the question, could the mitochondrion of cancer cells be evolved reversely and/or convergently to become a hydrogenosome or hydrogenosomal like organelle [62,63]? One of the supportive answers for this question is activity of metronidazole as radiosensitizer. Metronidazole is a pro-drug act against some protozoa and anaerobic bacteria. It is activated by low by PFO due to low redox potential and so it characterized by selective toxicity–harmful to parasite and not host- due to selective activation. During 1970s and early 1980s, metronidazole widely used as radio-sensitizer against hypoxic and/or anoxic core cells; do those type of cells possess hydrogenosome, PFO? If not, so how does cancer cell activate metronidazole [64-70]? Moreover, depletion and deletion of mitochondrial DNA cancer cells [71] might be additional support evidence for this suggestion because hydrogenosomes are organelles free genome with exceptions [72] and some of these exceptions could be a missed link between hydrogenosome and mitochondrion [73]. Interestingly, production of energy inside hydrogenosome is closed to that of mitochondrion that partially functional of glutaminolysis.

There are some evidences suggesting that there is a metabolic plasticity between the Warburg-effect and very low activity of Krebs cycle in certain tumors [74] and so central dogma of tumor metabolism become challenged [10]. Cancer cells have versatility of metabolism comes through diversity of strategies remove of mitochondrion, re-activation of complete mitochondrion and sometimes in between. Therefore, cancer metabolism characterized by plasticity and versatility through several diversified strategies. Some rules that might govern such occurrence of strategy:

- Location of cancer cells from blood vessels (if at more distance it means survive in hypoxia and then mitochondrial atrophy), if at the core, means anoxic and so means cannibalism. At edge, means oxygenated environment.
- Stage of tumors, metastases means cannibalism while early developed tumor means Warburg-effect and Crabtree-effect. Next question will be: What kind of metabolic strategy that has been carried when metastasized cell upon its settlement at distal site?
- Types of tissue: Although heart has highest mitochondrial number yet it has the lowest tendency to develop tumor while epithelium cells more vulnerable to develop cancer. Thus, mitochondrial number has inversely proportional to develop tumor. In contrast, epithelial have ton of mitochondria yet they undergo carcinogenesis. So, it is conundrum! Faint light that may shed on this mystery is functionality of mitochondrion e.g., Heart muscle during all life, have a great deal of mitochondria specialized only for energy production for and not on death pathway (this mainly upon ischemia heart cell undergo necrosis nor apoptosis). Based on this assumption, do mitochondria cross over the body follow tissue distribution and specialization? E.g., in heart mitochondrion responsible for energy production while that of skeletal muscle have less efficiency in energy production in compare to heart and replication tissue mitochondria might be specialized to be vulnerable and well prepared to be machinery for cellular death (in case of irreparable damage) rather than energy producing organelle? Taking heart cells as model to study why it is hard to develop cancer will enrich the filed by a great deal of valuable information. Parallel to the context, Warburg-Opinion that tumorigenesis occurs after metabolic injury should be re-appraisal. Interestingly, although heart muscle might never survive in hypoxia and so it is very rare to express Hypoxic proteins, so focusing on tumor hypoxia might draw us into astray way in tumor biology because brain does not tolerate hypoxia as well as heard yet it develops cancer.
- Do platelets undergo carcinogenesis although they contain mitochondria with lacking nucleus (especially upon recent evidence that platelets undergo cellular division) [75]? Based on this evidence, central dogma of cell division might be re-conceptualized again as nucleus represents central point on it? Interestingly, through endosymbiosis point of view, platelet represents trivial cell that lack host (nucleus) in presence of mitochondrion. Next question, will be do exosomal biogenesis might be considered as a symmetry cellular division?

## 6. Lactate Symbiosis

Upon reappraisal of Lactate-symbiosis [48], we might predict new way of metabolic thinking, Core of tumor is hypoxic and so it relies on glycolysis while edge of tumor is mainly in oxidative. So, lactate-symbiosis reflects tumor colony as integrated metabolic ecosystem. Likewise, we expect presence of isthmus to prevent of transgress between two types of metabolism systems or at least neutral area that possess semi-equifunctional glycolysis with Oxidative phosphorylation (OXPH). In addition, edge of tumor also relies on lactate as energetic fuel that produced from stroma [52] (see Figure 5). And the next question will be:

- What about pH gradient pH*i*/pH*e* and electrolytes distribution among oxidative phenotypes in compare to glycolytic phenotypes?
- What about nuclear-mitochondrial interaction in both types of cells Oxidative (at edge) and Warburg-phenotypes (at core)?
- Do oxidative phenotypes of cells are more vulnerable to undergo apoptosis in compare to glycolytic phenotypes, because they contain active mitochondria?
- Does, this strategy useful as a way of maintains pH at certain threshold and so it might prevent the excessive cell death [76]?
- Based on Reshkin pathway of Carcinogenesis, Do oxidative metabolic types of cells suitable habitats for Human Papilloma Virus?
- What are benefits of lactate-symbiosis (tumor cooperation)?
- Do mitochondria of the core are atrophied or translocate from the core into edge? If so, tumor colony looks-like only one giant multinucleated cell contains mitochondria at surface of it.
- Does this division of labor occur through quorum sensing [77] or another signaling mechanism?
- Does lactate-symbiosis limit metastasis formation? In contrast to this question, recent evidences suggest lactate production has great impact in angiogenesis [78].
- Does lactate-symbiosis occur in some types of leukemia?
- What happens for lactate-symbiosis if tumor colony undergoes further reverse and/or convergent evolution of glycolytic phenotypes under abstinent conditions into cannibal cells? If so, thus oxidative types of cells are more resistant to develop cannibalism because they are slightly glucose-independent types of cells and so Warburg-phenotypes are more vulnerable to acquire cannibalism as a compensation of glucose starvation. But still a remaining enigma how does these types of cells are independent of glucose; it is very interesting to find normal mammalian cells are independent of glucose.
- Lactate is a fuel for carcinogenesis, how does exogenous lactate induces spontaneous regression of cancer [24]?

Normal cells provided by both glucose and oxygen simultaneously. In hypoxia with glucose, it Pasteur/Crabtree-effects transform into and finally Warburg-effect. While absence of both glucose and oxygen develops cannibalism (Table 2). Interestingly, what happens in oxygenated environment in absence of glucose? It appears new metabolic pathway, oxidative without glycolysis. So, at this event cancer cell might represent an advanced generations during eukaryogenesis *i.e.*, extra-endosymbiosis.

## 7. Conclusions

We propose that carcinogenesis (malignant transformation) is a reverse evolution process as a way of resistance or at least delay cellular death process. This reversion is accompanied by metabolic transformation that occurs through series of sequential steps (cascade). Proper understanding of tumor metabolic will not enrich only oncology discipline but it will represent potential anticancer strategies.

## Figures and Tables

**Table 1.** Definitions of some scientific terms used in this review.

**Anti-Dollo's law:** In 1893 Louis Dollo pointed out that evolution is an irreversible process, *i.e.*, evolution is unidirectional. However, Anti-Dollo's law indicates flexibility of evolution suggesting the possibility of both reverse evolution and convergent evolution.
**Reverse Evolution (Atavism):** In common parlance, “devolution”, “de-evolution”, or backward evolution is the notion that a species can change into a more “primitive” form. Returning to ancestral state doesn't mean degeneracy but loss of more complex features can reflect adaptation to new environment. So, it is neither reverse evolution but it is adaptive evolution e.g., Cave dwelling animals.
**Convergent Evolution:** describes the acquisition of the same biological trait in unrelated lineages.
**Reshkin Pathway of Carcinogenesis:** the data that came out of the papers published by the Reshkin group demonstrated that the alkalinazation produced by the up-regulation of the Na^+^/H^+^ exchanger, NHE1, during neoplastic transformation of a normal cell drives the first alterations of the cells toward glycolytic metabolism in the presence of oxygen (Warburg effect) and the first appearances of the ‘hallmarks’ of carcinogenesis. That is, it defines the link between altered pH dynamics and the first pre-cancer steps.
**Gatenby Pathway of Carcinogenesis:** the group of Gatenby and Gilles has also demonstrated a link between pH and progression but at later phases of carcinogenic progression: when hypoxia, frequently encountered in solid tumors, forces tumor cells to perform anaerobic metabolism which as explained in this review results in an acid load. The tumor cells cope with the resulting acid load by extruding the excess protons via the Na^+^/H^+^ exchanger NHE1, which further acidifies the tumor environment and drives metastatic progression in a positive feedback loop. These two factors (hypoxia & acidic pH*e*) together with low nutrients due to the chaotic vasculature define the tumor metabolic microenvironment.
**Warburg-Effect:** 1n 1920s Otto Warburg discovered that tumor cells rely have higher fermentation rate. In other words, mainly it relays on glycolysis rather Krebs' cycle. In 2008, Pierre Sonveaux *et al.* discovered that not all tumor cells undergo Warburg-effect; tumor at edge of colony rely on lactate that produced due to Warburg-Effect in a phenomena termed as **Lactate-Symbiosis**. In 2009, Stephanos Pavlides *et al.* discovered that epithelial cancer cells induce the Warburg effect (aerobic glycolysis) in neighboring stromal fibroblasts. They stated … “In this scenario, the epithelial tumor cells “corrupt” the normal stroma, turning it into a factory for the production of energy-rich metabolites”… this phenomenon termed as **The Reverse of Warburg Effect**.

**Table 2.** Suppose oxygen reflects Krebs' cycle and Glucose reflects glycolysis. Warburg-Effect comes through either adaptation to hypoxia (Gatenby Pathway) or direct activation of glycolysis (Reshkin Pathway). Parallel to the context, Lactate-symbiosis might occur either through direct activation of Krebs' cycle due to acidifying of intracellular pH (pH*i*) [5] and/ or compensation of glycolysis inhibition.

**Type of Metabolism**	**Glycolysis**	**Krebs' Cycle**
Normal Metabolism	+	+
Warburg Effect	+	−
Lactate Symbiosis/Reverse of Warburg-effect	−	+
Cannibalism	−	−
